# Genotype × Environment Effects in Three Wild Relatives of Sorghum From Australia

**DOI:** 10.1002/pei3.70065

**Published:** 2025-06-06

**Authors:** Harry Myrans, Dinithi Chithrarachchige, Robert J. Henry, Sally Norton, Roslyn M. Gleadow

**Affiliations:** ^1^ School of Biological Sciences Monash University Melbourne Victoria Australia; ^2^ Institute of Climate, Energy and Disaster Solutions The Australian National University Canberra Australian Capital Territory Australia; ^3^ Queensland Alliance for Agriculture and Food Innovation The University of Queensland St Lucia Queensland Australia; ^4^ Australian Grains Genebank Agriculture Victoria Horsham Victoria Australia

**Keywords:** aridity, Australian native plants, crop wild relatives, drought, genotype × environment interaction, plant breeding, water stress

## Abstract

Endemic wild *Sorghum* species are prevalent across northern Australia and could be useful for crop improvement; however, few studies have been done to quantify the phenotypic diversity of this tertiary gene pool. We aimed to assess the interactive effects of genotype and water availability in three wild *Sorghum* species native to northern Australia and compare these to domesticated sorghum ( *Sorghum bicolor* ). Two accessions of wild *Sorghum plumosum*, *Sorghum stipoideum* , and *Sorghum timorense*, sourced from more and less arid regions, were grown alongside a *S. bicolor* line under well‐watered or drought conditions for 4 weeks. We measured biomass, root:shoot ratio, chlorophyll *a*:*b* ratio, and concentrations of chlorophyll. The concentration of phenolics and cyanogenic glucosides were also measured to see if there were any differences in the concentration of specialized metabolites, as this is of particular importance for grazing. Low soil moisture (“drought”) significantly impacted the biomass, root:shoot ratio, and chemical composition of *S. bicolor* , but the effects on the wild accessions were minimal and mostly not significant. This is potentially a consequence of their adaptation to harsh conditions in northern Australia. In each of the wild study species, genotype effects (i.e., between accessions) were greater than treatment effects, indicating intraspecific diversity. Wild *Sorghum* is a potential source of novel traits that could be helpful in further enhancing the ability of *S. bicolor* to tolerate hot and dry conditions. Further research into traits conferring drought tolerance in *Sorghum* without compromising yield is needed.

## Graphical abstract

Growth, dhurrin, and phenolic concentrations of three species of wild *Sorghum* native to Australia grown under well‐watered and water‐limited conditions varied more with genotype (i.e., among species and the aridity of the source populations) than experimental treatments.

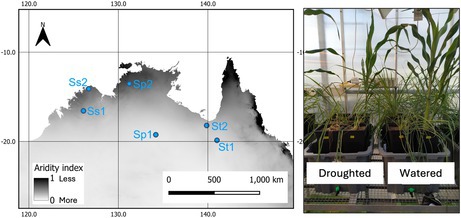

## 1 Introduction

*Sorghum* Moench is a diverse genus of plants best known for the domesticated crop, sorghum ( *Sorghum bicolor* [L.] Moench). Sorghum was domesticated around 5000 years ago in Africa, and today it is grown globally for diverse products including grain, feed, fodder, and fuel (Fuller and Stevens 2018). Despite originating in Africa, most of sorghum's wild congeners are native to northern Australia, where they form an important part of the understorey (Lazarides et al. 1991; Myrans et al. 2020). In northern Australia, plants face harsh conditions such as fire, high temperatures, poor soils, and low rainfall (Andrew and Mott 1983; Bowman et al. 2010). These conditions exert strong selection pressures on plants, shaping their evolution and phenotypic traits. Over the vast area of northern Australia, each of these conditions varies considerably, meaning that phenotypes of wild *Sorghum* populations can greatly differ, even within species (Dillon et al. 2007; Myrans et al. 2020). *Sorghum* is, therefore, a good model genus for studying intraspecific and interspecific trait diversity, including between wild and domesticated species. However, what little is currently known has generally been established using a handful of accessions (Cowan et al. 2020, 2022; Myrans et al. 2021). Further quantification of the phenotypic diversity in the gene pool within *Sorghum* is needed. Such work may uncover adaptations that could be transgressed into domesticated sorghum to improve tolerance to the increasingly hostile global conditions for agriculture (Ananda et al. 2020).

In order to quantify intraspecific diversity, three study types are typically implemented: cline studies, field observations, and genotype × environment (G × E) interaction studies (Carvalho et al. 2022). Myrans et al. (2024) recently carried out a cline study on three Australian *Sorghum* species (*Sorghum plumosum* [R.Br.] P.Beauv., *Sorghum stipoideum* [Ewart & Jean White] C.A.Gardner & C.E.Hubb. and *Sorghum timorense* [Kunth] Büse), identifying phenotypic differences among populations found along aridity clines. They found evidence of intraspecific variation among populations, with significant variation found in 8 of 10 traits measured in *S. plumosum* . However, no trait consistently correlated with aridity across all three study species. It was suggested that the interactive effects of genotype and the environment may more clearly uncover the adaptations of populations to the aridity of their provenance.

We aimed to continue quantifying the intraspecific diversity of *S. plumosum* , *S. stipoideum* , and *S. timorense* by carrying out a G × E study in a controlled environment. G × E studies can either take place in the field or controlled environments. Field studies, such as reciprocal transplant experiments, are lauded for their realism because they expose study plants to the true conditions faced by each population (Kawecki and Ebert 2004). Here, a controlled environment was used, allowing us to test the effect of a single environmental stimulus without confounding variables. Water availability was selected as the environmental stimulus. An improved *S. bicolor* line was included to evaluate the efficacy of the method and compare tolerance and plasticity. Traits selected for this study were biomass, root:shoot ratio, chlorophyll concentration, and chlorophyll *a*:*b* ratio, as well as leaf phenolic and cyanogenic glucoside concentrations, all of which are commonly influenced by water availability (Gleadow et al. 2016; Iqbal et al. 2020; Keyvan 2010; Wilson 1988).

Cyanogenic glucosides are stable, nitrogen‐containing secondary metabolites found in more than 10% of plant species (Gleadow and Møller 2014; Wang et al. 2024). Herbivory causes cyanogenic glucosides to be hydrolysed by facilitating mixing with specific bioactivating β‐glucosidases, releasing toxic hydrogen cyanide (HCN) in a process named cyanogenesis. Cyanogenic glucosides have long been assumed to serve as a defense against generalist herbivores (Bak et al. 2006; Gleadow and Møller 2014; Zagrobelny et al. 2008). However, there is evidence that cyanogenic glucosides can also serve other functions, including as a storage molecule for reduced nitrogen (Blomstedt et al. 2018; Møller 2010). The cyanogenic glucoside in all species of *Sorghum*, including the Australian wild relatives, is dhurrin (Cowan et al. 2022). In domesticated sorghum, dhurrin concentrations typically increase under drought conditions (Cowan et al. 2020; Gleadow et al. 2016; O'Donnell et al. 2013). However, there is no consistent evidence in the more distantly related wild *Sorghum* species that dhurrin biosynthesis is up‐regulated during drought or other stress, with studies showing variously an increase, a decrease, or no change, depending on the species (Ananda et al. 2022; Cowan et al. 2020). Only a single accession of a handful of species was included in these experiments, so it is possible that environmental (E) effects have simply not been detected. The only study comparing genotype effects (G) across multiple accessions grew the plants in a well‐watered common garden (Myrans et al. 2024). By testing G × E interaction effects, we aim to assess whether up‐regulation of dhurrin in response to drought is population‐specific or absent from wild *Sorghum* more generally.

The aim of this study was to determine how the phenotypes of the little studied wild *Sorghum* species native to Australia vary under the interactive effects of genotype and growing conditions. We included two accessions of three wild *Sorghum* species ( *S. plumosum* , *S. stipoideum* and *S. timorense*) sourced from provenances of varying aridity levels, as well as one line of *S. bicolor* for comparison. Plants were grown in a greenhouse for 8 weeks in total (i.e., 10 weeks from germination): 4 weeks under a well‐watered (control) treatment or a water‐limited (drought) treatment. Our hypotheses were: (1) within accessions, droughted plants would have slower growth and higher concentrations of dhurrin and phenolics than control plants; (2) within wild species, accessions from more arid provenances would have more plastic traits than accessions from less arid provenances; and (3) that relative to control plants, domesticated sorghum would be more impacted by the drought treatment than the wild accessions.

## 2 Materials and Methods

### 2.1 Plant Material and Growth Conditions

**FIGURE 1:**
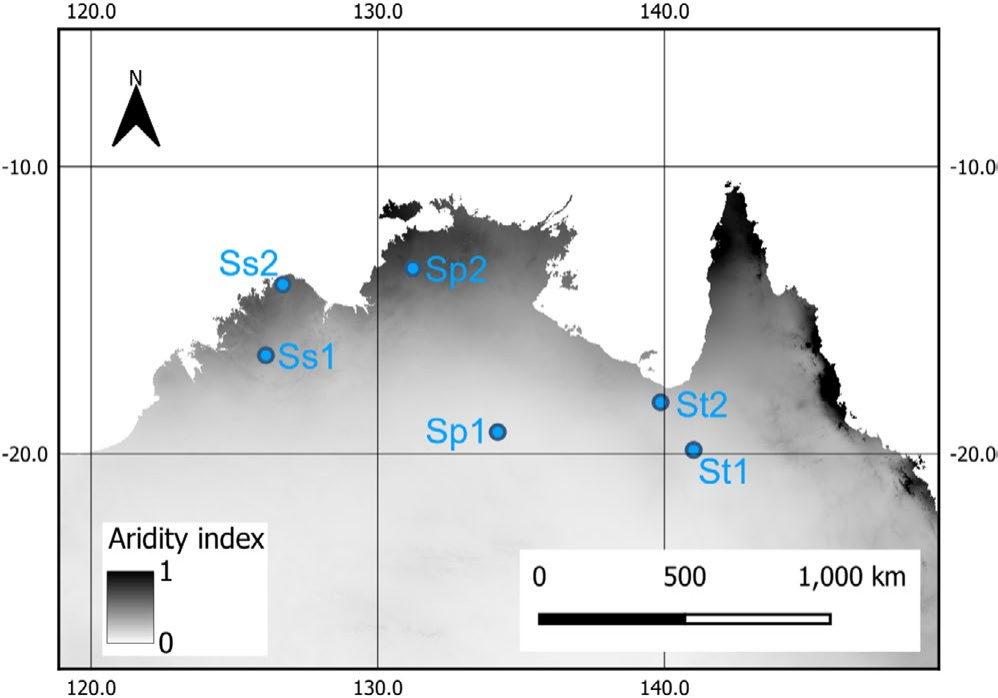
Map of northern Australia showing collection sites for two populations of the three species of wild sorghum used in this study (blue circles): Sp, *S. plumosum* ; Ss, *S. stipoideum* ; St, *S. timorense*. Accession 1 of each species was collected from a more arid part of its range and accession 2 collected from a less arid region. Aridity indices were calculated according to Williams et al. (2010), with high values indicating less arid areas (black) and low values (white) indicating more arid areas (see Table 1).

Two *S. plumosum* accessions, two *S. stipoideum* accessions, and two *S. timorense* accessions were germinated and grown in a standardized greenhouse environment for 8 weeks. The study accessions are a subset of those studied by Myrans et al. (2024) and sourced from either a more (1) or less (2) arid provenance, as defined by Williams et al. (2010) (Figure 1; Table 1). A well‐studied, improved *S. bicolor* line, BTx623, was grown as the domesticated genotype for comparison.

**TABLE 1:** Coordinates of the collection sites and corresponding aridity indices for the seven accessions across the *Sorghum* species examined in this study ( *S. plumosum* , *S. stipoideum* , *S. timorense* and *S. bicolor* ).

Accession code	Species	Accession number^a^	DOI	Accession name	Latitude	Longitude	Temperature (°C)^b^	Annual rainfall (mm)^b^	Aridity index^c^
Sp1	*S. plumosum*	AGG 302635 WSOR	10.18730/1E772G	JC 2306	−19.2485	134.1803	25.96	411	0.109
Sp2	*S. plumosum*	AGG 302469 WSOR	10.18730/1E6DNH	JC 2114	−13.5453	131.2220	26.99	1302	0.676
Ss1	*S. stipoideum*	AGG 302664 WSOR	10.18730/18A4PS	JC 2347	−16.5725	126.0928	25.70	849	0.358
Ss2	*S. stipoideum*	AGG 302621 WSOR	10.18730/18A458	JC 2356	−14.1090	126.6820	27.62	1125	0.589
St1	*S. timorense*	AGG 302532 WSOR	10.18730/1E6HS1	JC 2209	−19.8732	141.0175	26.27	496	0.160
St2	*S. timorense*	AGG 302500 WSOR	10.18730/1E6FQ9	JC 2159	−18.2123	139.8640	26.85	604	0.242
Sb	*S. bicolor*	N/A		BTx623	N/A	N/A	N/A	N/A	N/A

^a^ Australian Grains Genebank accession number (https://ausgenebank.agriculture.vic.gov.au/gringlobal/).

^b^ Mean annual temperature and rainfall according to Fick and Hijmans (2017).

^c^ Mean annual aridity index according to Williams et al. (2010).

Seeds of wild accessions were provided by the Australian Grains Genebank (Horsham, Australia), collected by the Australian Tropical Crops Genetic Resource Centre. All plants (*n* = 90) were grown in a greenhouse at Monash University (−37.91°, 145.14°) between February and April 2023, with a mean temperature of 33.29°C ± 0.07°C and 24.17°C ± 0.04°C day/night, a mean photoperiod of 12 h, and a mean relative humidity of 49.3% ± 0.2%. Supplementary light was provided using sodium lamps for 13 h per day (MK‐1 Just‐a‐shade; Ablite).

Seeds were germinated following Cowan et al. (2020). Once the coleoptile and radicle had emerged, seedlings were planted in seedling trays containing Debco Seed and Cutting Premium Germinating Mix (Evergreen Garden Care Australia) and river sand (Bastion Pacific Pty Ltd) (2:1 v/v ratio) for 2 weeks. Seedlings were then transplanted into 6 L pots containing the same substrate, with 30 g Scotts Osmocote All Purpose Landscape Controlled Release Fertilizer (Evergreen Garden Care Australia) added. Each pot had four 51 mm holes drilled into its base and was lined with 20 cm^2^ of 20 μm nylon mesh (Allied Filter Fabrics).

**FIGURE 2:**
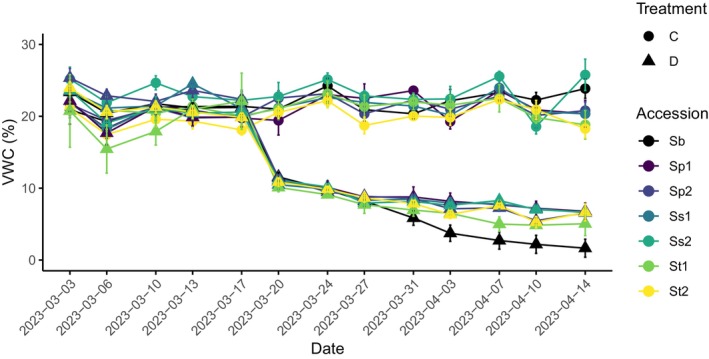
Soil volumetric water content (VWC ± SEM), in control (C, gray circles) pots and drought (D, black triangles) measured twice per week during plant growth. The accession is indicated by color (see Table 1). Each of the wild species (Sp, *S. plumosum* ; Ss, *S. stipoideum* ; St, *S. timorense*) were sourced from more (1) or less arid (2) regions of the northern Australia. Sb, *S. bicolor* . Drought treatment began on 17 March 2023.

Control and drought treatments were imposed following Marchin et al. (2020). The potted plants were placed on 23 cm Ideal Floral Foam Maxlife Bricks (OASIS Floral Australia), which were standing inside 100 L plastic tubs filled with water to 1 cm below the top of the floral foam (*z* = 1 cm). There were 16 tubs in total (eight per treatment), with five or six plants per tub (organized in randomized incomplete blocks). For the first 2 weeks after transplanting, plants were allowed to adapt to capillary watering in the tubs, with all tubs at *z* = 1 cm. After this, half the tubs stayed at *z* = 1 cm (control treatment), and the other half were drained until *z* = 21 cm (drought treatment) for 4 weeks, that is, 8 weeks after transplanting. This duration was chosen as it had been shown by Rosati, Blomstedt, et al. (2019) to be sufficient to induce changes in composition and biomass, without causing extensive senescence or death. We also wanted to complete the experiment when plants were still in the vegetative stage as the time to flowering is unknown in the wild species. Tub water levels were topped up twice per week to maintain consistent *z* levels. Soil volumetric water content (VWC; %) of each pot was measured twice weekly using a CS658 HydroSenseII with a 20 cm water content probe (Campbell Scientific Inc.). If VWC dropped below 20% or 5% in control or drought pots, respectively, the pot was top‐watered with 250 mL water to maintain target VWC (Figure 2). Replicate numbers for each group at the start and end of the treatment period can be found in Table 2.

**TABLE 2:** Number of replicates at the start and end of the treatment period for each study accession.

Accession	Replicates at start of control treatment	Replicates at end of control treatment	Replicates at start of drought treatment	Replicates at end of drought treatment
Sp1	4	4	4	4
Sp2	7	7	7	7
Ss1	7	7	7	5
Ss2	7	6	7	7
St1	7	6	7	2
St2	7	7	7	7
Sb	6	6	6	6

*Note:* Accessions are labeled according to Table 1.

### 2.2 Harvesting and Growth Analysis

Plants were harvested 4 weeks after the imposition of the treatment (i.e., 8 weeks after transplanting). Plants were separated into leaves (leaf blades), sheaths (leaf sheaths plus stems), and roots, and oven dried at 50°C for 72 h. Plant growth was quantified by measuring the biomass of the leaves, sheaths, and roots for each plant. Senesced leaves were included in total biomass measurements, but not in chemical analysis. The root:shoot ratio was calculated using the following equation:

$$\text{Root}:\text{shoot ratio}=\frac{\text{root biomass}}{\left(\text{leaf biomass}+\text{sheath biomass}\right)}$$

### 2.3 Chlorophyll and Phenolics

Concentrations of chlorophyll *a* and *b* were measured following Gleadow et al. (1983), as modified by Burns et al. (2002). Chlorophyll was extracted from 10 mg dried, ground leaf tissue by mixing with 1.5 mL 80% acetone solution and a ball bearing before shaking in a TissueLyser II (Qiagen) for 1 min. The mixture was centrifuged at 15,000 rpm for 2 min and the supernatant collected. The pellet was then twice resuspended in 0.5 mL 80% acetone, shaken for one more minute, and centrifuged for 2 min, with supernatants from the three extractions per sample pooled. The pooled supernatant was poured into a 1.6 mL semi‐micro cuvette and its absorbance was measured at 664 and 647 nm in a Cary 50 Bio UV–Visible Spectrophotometer (Varian).

Phenolic concentration was measured by colorimetric assay in 96‐well microtiter plates, following a Folin–Ciocalteu method adapted from Ainsworth and Gillespie (2007) and Bärlocher and Graça (2020). Ten milligrams of ground tissue was mixed with 1 mL 70% acetone solution. Twenty microliters of acetone mixture was then mixed with 20 μL distilled water, 20 μL diluted Folin–Ciocalteu reagent, and 200 μL of 2% sodium carbonate in 0.1 M sodium hydroxide solution. This mixture was incubated at room temperature for 2 h before its absorbance was measured at 760 nm (FLUOstar Galaxy; BMG Labtechnologies). Gallic acid (91215; Sigma‐Aldrich) was used to produce the standard curve.

### 2.4 Cyanogenic Glucosides

Dhurrin concentration in dried tissue was measured by determining HCN potential‐varied with the total amount of HCN released from plant tissue following Woodrow et al. (2002). Ten milligrams of dried, ground tissue was mixed with 300 μL of β‐glucosidase solution within a sealed vial to catabolize all its CNglcs and release gaseous HCN. The HCN was trapped in 200 μL 1 M sodium hydroxide solution in a microcentrifuge tube inside the sealed vial to form sodium cyanide, the concentration of which was then quantified through König reactions followed by measuring absorbance at 590 nm (FLUOstar Galaxy; BMG Labtechnologies). Sodium cyanide (380970; Sigma‐Aldrich) was used as the standard.

### 2.5 Statistical Analysis

Statistical analyses for phenotypic parameters were performed in GraphPad Prism 9.0.1 (GraphPad Software) and R version 4.3.2 (R Core Team 2023). For each parameter, data were separated by species. For each phenotypic parameter, data were separated by species. Within *S. bicolor* , biomass data were analyzed using two‐tailed *t*‐tests. For all other parameters, analysis of covariance (ANCOVA) was performed to assess treatment effects while controlling for biomass. Within *S. plumosum* , *S. stipoideum* , and *S. timorense*, biomass data were analyzed using two‐way ANOVAs. For all other parameters, ANCOVAs were performed to assess the effects of treatment and genotype while controlling for biomass. For all analyses, *p* < 0.05 was considered significant. Where necessary, data were log‐transformed to reduce skewness. If the treatment × biomass effect in an ANCOVA was significant, separate slopes analysis was performed. VWC data were separated into accession × treatment groups and analyzed by two‐way ANOVA. If significant effects were found (*p* < 0.05), Tukey's tests were performed (Table S1). Raw data are available online (Gleadow et al. 2025).

## 3 Results

### 3.1 Growth Parameters

**FIGURE 3:**
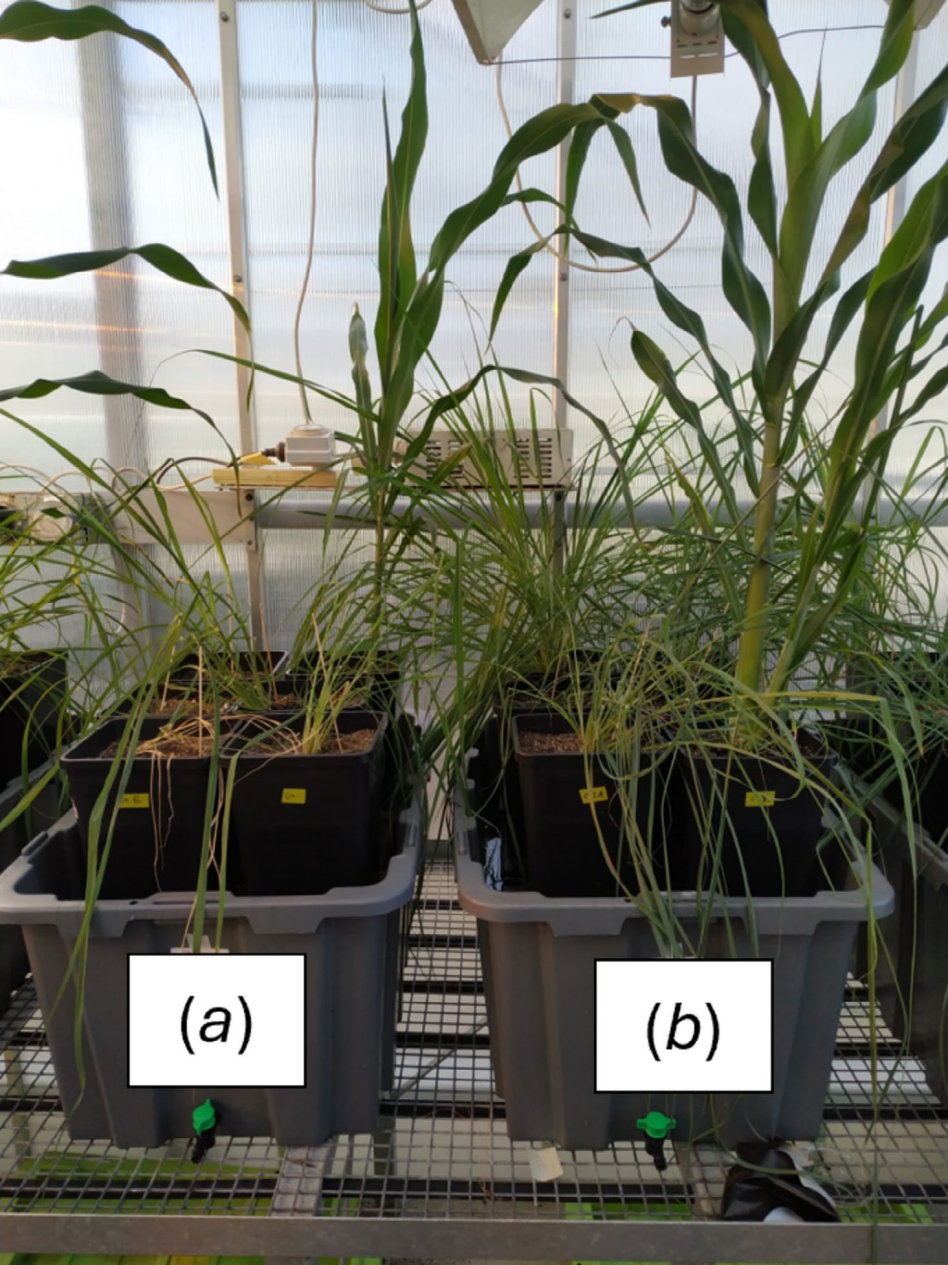
Ten‐week‐old study plants after 4 weeks of growth under an (a) drought or (b) control treatment. Each tub contained 5–6 plants. The degree of water stress was controlled by the level of water in the tub (see Figure 2).

**FIGURE 4:**
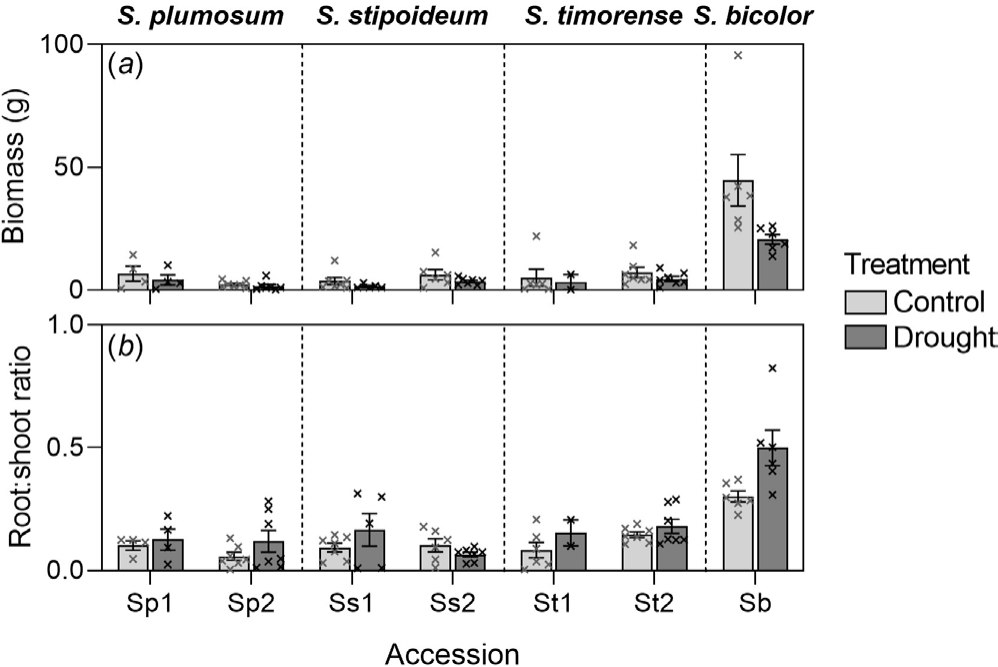
(a) Biomass and (b) root:shoot ratio in seven *Sorghum* accessions: Two accessions of three wild species of *Sorghum* from northern Australia ( *S. plumosum* , *S. stipoideum* and *S. timorense*) and *S. bicolor* . Plants were 10 weeks old and had been grown in a control or drought treatment for 4 weeks. Accessions numbered “1” were from more arid provenances than those labeled “2” (see Table 1). Bars represent means ± SEM of 2–7 replicates. Crosses represent individual datapoints.

Overall, biomass was much higher in domesticated *S. bicolor* than in the wild species, with the biomass of wild accessions ranging from 1.6 to 7.2 g, among species and treatments. In *S. bicolor* , mean biomass was more than double under the control treatment (44.7 g) than under the drought treatment (20.7 g) (*p* < 0.05) (Figures 3 and 4). The effect of treatment on root:shoot ratio varied significantly depending on biomass. Increases in root:shoot ratio, relative to increased biomass, were significantly greater under drought conditions than under the control (Figure 4; Table S2; Figure S1a).

In most accessions, all individuals survived the drought treatment, except for Ss1, of which five of seven replicates survived, and St1, of which only two of seven replicates survived. Within *S. plumosum* and *S. timorense*, there were no significant differences in biomass among accessions or treatments. In *S. stipoideum* , biomass was significantly higher overall in accession Ss2 than in Ss1. In all wild accessions, mean biomass was higher in control plants than in drought plants, but differences were not significant. Within the wild species, no significant differences in root:shoot ratio among accessions or treatments were found (Table 3; Figure 4; Tables S3–S6).

**TABLE 3:** Results of two‐way ANCOVAs testing effects of genotype, treatment, biomass, and their interaction on phenotypic traits in *S. plumosum* , *S. stipoideum* , *S. timorense*, *and S. bicolor*.

Species	Parameter	Effect					
		Genotype	Biomass	Treatment	Genotype × biomass	Biomass × treatment	Genotype × treatment
*S. plumosum*	Root:shoot ratio	0.528	0.224	0.098	0.320	0.916	0.503
	Total [chlorophyll]	0.987	0.297	0.308	0.180	0.987	0.220
	Chlorophyll *a*:*b* ratio^a^	0.384	0.219	0.148	0.201	0.724	0.068
	Leaf [phenolics]	**< 0.001**	0.458	0.715	0.076	0.479	0.140
	Leaf HCN potential^a^	**0.002**	0.713	0.338	0.629	0.388	0.489
	Sheath HCN potential^a^	**0.049**	0.185	0.686	0.405	0.922	0.276
	Root HCN potential^a^	**0.015**	0.820	0.510	0.996	0.598	0.793
*S. stipoideum*	Root:shoot ratio^a^	0.229	0.368	0.458	0.502	0.868	0.141
	Total [chlorophyll]	0.829	**0.039**	0.063	0.569	0.983	0.782
	Chlorophyll *a*:*b* ratio^a^	0.455	0.275	0.707	0.331	0.624	0.422
	Leaf [phenolics]	0.425	0.461	**0.032**	0.569	0.192	0.441
	Leaf HCN potential^a^	**0.020**	0.391	0.647	0.448	0.465	0.624
	Sheath HCN potential^a^	0.087	0.832	0.736	0.669	0.329	0.686
	Root HCN potential^a^	0.454	0.417	0.722	0.504	0.616	0.454
*S. timorense*	Root:shoot ratio	0.055	0.654	0.121	0.863	0.354	0.448
	Total [chlorophyll]	0.190	0.737	0.822	0.161	0.900	0.371
	Chlorophyll *a*:*b* ratio	0.531	0.513	0.588	0.107	0.748	0.900
	Leaf [phenolics]	0.245	0.380	0.222	0.313	0.151	0.173
	Leaf HCN potential^a^	0.552	0.348	0.844	0.539	0.281	0.849
	Sheath HCN potential^a^	**< 0.001**	0.496	0.075	0.136	**0.040**	0.850
	Root HCN potential	0.648	0.761	0.452	0.899	0.413	0.769
*S. bicolor*	Root:shoot ratio	N/A	0.100	**0.003**	N/A	**0.006**	N/A
	Total [chlorophyll]	N/A	0.183	**0.013**	N/A	0.068	N/A
	Chlorophyll *a*:*b* ratio	N/A	0.224	**0.003**	N/A	**0.005**	N/A
	Leaf [phenolics]	N/A	0.141	**0.023**	N/A	**0.023**	N/A
	Leaf HCN potential	N/A	**0.025**	**0.024**	N/A	**0.004**	N/A
	Sheath HCN potential	N/A	0.898	**0.015**	N/A	0.083	N/A
	Root HCN potential^a^	N/A	0.063	0.752	N/A	0.971	N/A

*Note:* *p* Values for each analysis are shown, with bolded values denoting significant results (*p* < 0.05). *n* = 2–7. Full ANCOVA results are shown in Tables S3–S6.

^a^ Data were log‐transformed for this parameter.

### 3.2 Chlorophyll and Phenolics

**FIGURE 5:**
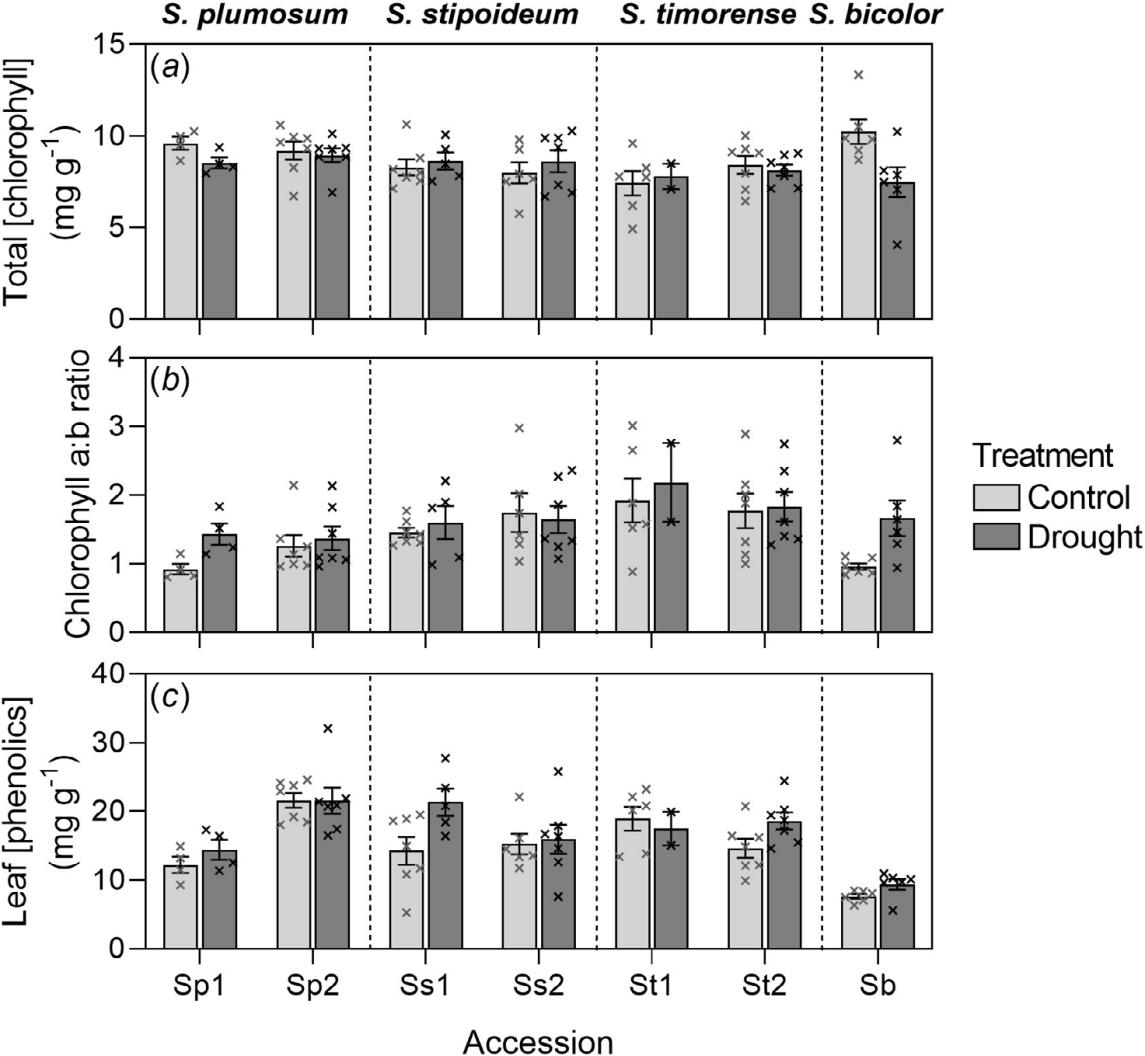
(a) Total chlorophyll content, (b) chlorophyll *a*:*b* ratio and (c) leaf phenolic concentration in seven *Sorghum* accessions ( *S. bicolor* and two accessions of each of *S. plumosum* , *S. stipoideum* and *S. timorense*). Plants were 10 weeks old and had been grown in a control or drought treatment for 4 weeks. Accessions numbered “1” were from more arid provenances than those labeled “2” (Table 1). Bars show means ± SEM of 2–7 replicates. Crosses represent individual datapoints.

*Sorghum bicolor* was the only species for which total chlorophyll concentration significantly varied with treatment, with control plants having a higher mean chlorophyll concentration (10.2 mg g^−1^) than drought plants (7.5 mg g^−1^) (Figure 5a). Chlorophyll *a*:*b* ratio also varied significantly with treatment in *S. bicolor* , with increases in chlorophyll *a*:*b* ratio, relative to increased biomass, significantly greater under drought conditions than the control (Figure S1b). In the wild species *S. stipoideum* and *S. timorense*, no significant differences in chlorophyll *a*:*b* ratio among accessions or treatments were found. In *S. bicolor* , increases in leaf phenolic concentration relative to increased biomass were significantly greater under drought conditions than the control (Figure S1c). In *S. plumosum* , mean leaf phenolic concentration was significantly higher in Sp2 than Sp1, while in *S. stipoideum* , the drought treatment significantly increased phenolic concentration. Phenolic concentrations in *S. timorense* were not significantly affected by treatment or genotype (Figure 5c; Table 3).

### 3.3 HCN Potential

**FIGURE 6:**
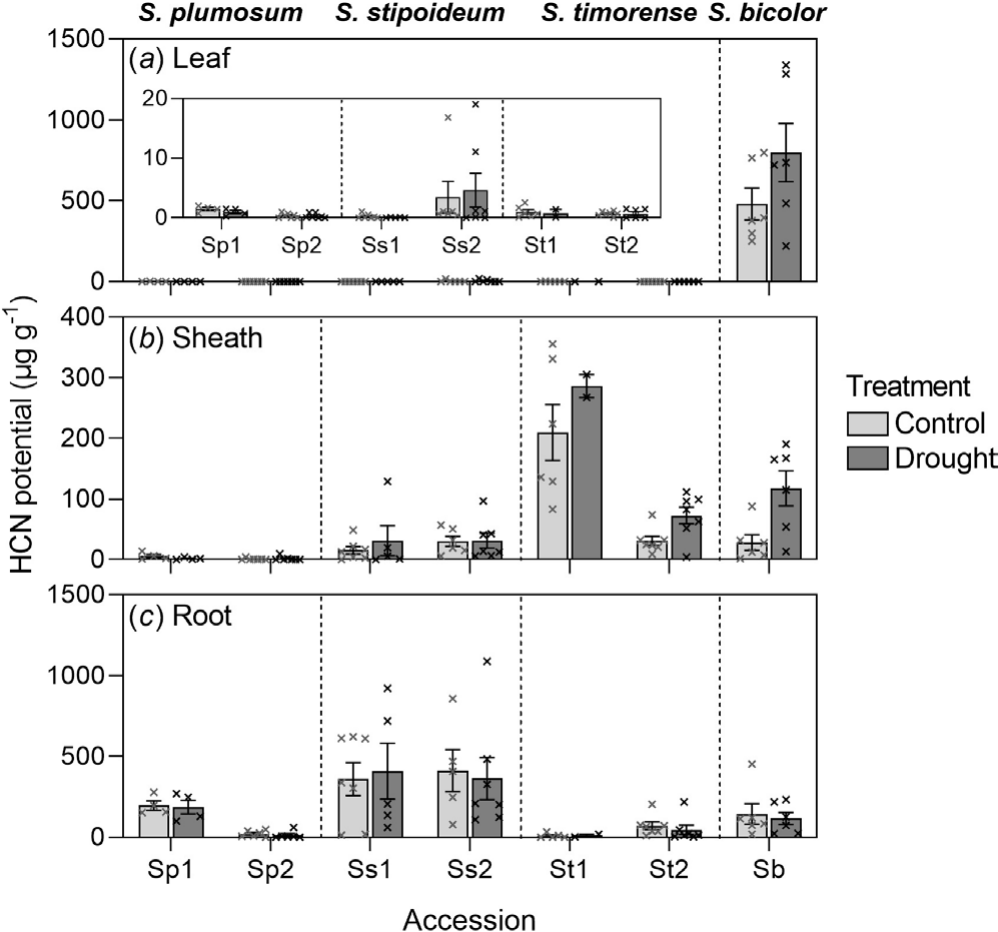
HCN potential of (a) leaves, (b) sheaths and (c) roots of *S. bicolor* and two accessions of each of *S. plumosum* , *S. stipoideum* , and *S. timorense*. Accessions numbered “1” were from more arid provenances than those labeled “2” (Table 1). Plants were 10 weeks old and had been grown in a control or drought treatment for 4 weeks. Bars show means ± SEM of 2–7 replicates. Crosses represent individual datapoints.

Overall, significant differences in dhurrin concentration (measured as HCN potential) were detected among accessions and species, and differed in different plant organs, but the effect of drought on HCN potential was only significant in *S. bicolor* (Figure 6). There were marked differences in HCN potential between aboveground and belowground concentrations irrespective of treatment. In *S. bicolor* , HCN potential was highest in the leaves under both treatments. HCN potential of the leaves in all wild species was negligible (Figure 6a). In *S. plumosum* and *S. stipoideum* , HCN potential was highest in the roots. In *S. timorense*, it was highest in the sheath (Figure 6).

In *S. bicolor* , treatment, biomass, and their interaction all significantly varied with leaf HCN potential. Levels were much higher in drought plants (797.5 μg g^−1^) than control plants (480.6 μg g^−1^). Mean leaf HCN potential was much higher in drought plants (797.5 μg g^−1^) than control plants (480.6 μg g^−1^). This difference was not significant at the 95% level, possibly due to the large degree of variability among individuals. Leaf HCN potential did not significantly vary with treatment or genotype within *S. timorense* (0.7–1.0 μg g^−1^). There were no treatment effects on HCN leaf potential in *S. plumosum* or *S. stipoideum* either. Interestingly, a significant genotype effect was detected in both these species (Table 3), with accessions Sp1 and Ss2 having higher leaf HCN potentials overall than Sp2 and Ss1, respectively (Figure 6a).

Sheath HCN potential was significantly higher in droughted *S. bicolor* compared with plants grown under control conditions. In *S. plumosum* , genotype had a significant effect on sheath HCN potential, with accession Sp1 having a higher mean sheath HCN potential than Sp2. In *S. timorense*, a significant interaction between biomass and treatment was detected, with HCN potential increasing with increased biomass under the drought treatment and decreasing with increased biomass under the control treatment (Figure S2). There were no significant effects found for sheath HCN potential in *S. stipoideum* (Table 3; Figure 6b).

There was no significant treatment effect on root HCN potential in any species. There was, however, a significant genotype effect detected in *S. plumosum* and *S. timorense*, with accessions Sp1 and St2 respectively having higher mean root HCN potentials than Sp2 and St1. Root HCN potential was similar across the two *S. stipoideum* accessions (Figure 6c).

## 4 Discussion

Australia's native sorghums are a potential source of novel traits that could be transgressed into cultivated genotypes to improve crop production (Ananda et al. 2020; Cowan et al. 2022). Here we compared the growth and chemical composition of three species ( *S. plumosum* [Sp], *S. timorense* [St] *and S. stipoideum* [Ss]) grown under well‐watered and water‐limited (drought) conditions and compared this to the widely cultivated *S. bicolor* (Sb). Two accessions of each species that had been sourced from populations growing in either more or less arid regions of their range in tropical northern Australia were included (Figure 1) (Myrans et al. 2020).

### 4.1 Survival and Growth

The high survival rate overall demonstrates the drought tolerance of both wild and domesticated *Sorghum* species, likely a result of adaptation to semi‐arid habitats (Dillon et al. 2007; Lazarides et al. 1991). However, the low survival rate of St1 under drought did create challenges for statistical analysis, especially through reducing the power available for detecting significant treatment effects within *S. timorense*.

It was unexpected that there would be no significant treatment effect on biomass or root:shoot ratio in any of the wild species. In contrast, droughted *S. bicolor* had a significantly lower biomass and a higher root:shoot ratio than control plants (Figure 4). All of these changes are commonly seen in drought‐stressed plants (Iqbal et al. 2020; Wilson 1988). Cowan et al. (2020) also found that drought did not significantly impact relative growth rate in six of the seven wild sorghum species that they tested, but it did significantly lower relative growth rate in *S. bicolor* , supporting our result. This could be due to greater drought tolerance in the wild species, or that the wild species did not experience the same degree of stress as a result of differences in plant architecture. It is noteworthy that in both our study and that of Cowan et al. (2020), mean biomass of the wild accessions was higher in control plants than drought plants, just not significantly. The slow growth rate of these species, compared to that of *S. bicolor* , might mean that more time is needed for treatment effects on growth to become significantly different. In comparison, a study by Quiroga et al. (2013) on the wild grass, *Trichloris crinita* (Lag.) Parodi, found significant interactive effects between provenance aridity and watering regime, with the more arid‐adapted population maintaining growth better than the less arid‐adapted one under a drought treatment. These *T. crinita* plants were grown for 6 months before treatments began, possibly allowing for easier identification of treatment effects on growth. The duration used in the present experiment was chosen to align with the work of Cowan et al. (2020), who did detect changes in composition in some species. However, the design of our experiment was such that the supply of water, whether replete or limited, was consistent unlike the pot weighing method used by Cowan et al. (2020), which allowed pots to dry between watering days.

The total chlorophyll concentration was significantly lower, and the chlorophyll a:b ratio was significantly higher in droughted *S. bicolor* than control plants, consistent with previous observations (Figure 5) (O'Donnell et al. 2013). In contrast, neither parameter was significantly affected by treatment in the wild species. This was somewhat unexpected, given that water stress has been shown to consistently impact these parameters (Keyvan 2010). While the wild species' ability to maintain chlorophyll concentrations may indicate superior drought tolerance (Li et al. 2006), it could also mean that the level of stress experienced by these plants was moderated by their narrow leaves and other aspects of their architecture.

### 4.2 Specialized Metabolites: Phenolics and Cyanogenic Glucosides

Leaf phenolic concentration was not significantly affected by treatment in any species (Figure 5c). Within *S. plumosum* , accession Sp2 had a significantly higher concentration than Sp1. This result matches that of Myrans et al. (2024), who also found a significantly higher leaf phenolic concentration in Sp2 than Sp1 (accessions P2 and P6 in that study, respectively). This result was somewhat unexpected given that phenolics can mitigate heat and drought stress and are therefore often viewed as a useful adaptation to arid environments (Carvalho et al. 2022). However, phenolics do represent a diverse range of molecules with multiple roles (Lattanzio et al. 2012). Thus, a single stimulus might cause some phenolics to accumulate and others to decumulate (Ahlawat et al. 2024), complicating the relationship of total phenolic concentration with provenance aridity and drought. Chromatographic methods that allow the separate measurement of different phenolics might allow us to better understand the importance of each compound to populations from different provenances and to plants experiencing different watering regimes.

*S. bicolor* stores most of its dhurrin in the aboveground parts of the plant, likely serving as a defense against herbivory in this species (Cowan et al. 2021). By contrast, in the present study, HCN potential in *S. plumosum* and *S. stipoideum* was higher in the roots than in any other organ (Figure 6). This has been observed in wild species before: in multiple accessions of *S. plumosum* and *S. stipoideum* (Myrans et al. 2024), as well as in single accessions of *Sorghum brachypodum*, *Sorghum intrans*, and *Sorghum macrospermum* (Cowan et al. 2020; Myrans and Gleadow 2022; Myrans et al. 2021). Storage of nitrogenous dhurrin in roots possibly safeguards some of the plant's nitrogen stores from herbivory and fire, allowing regrowth after aboveground losses (Myrans et al. 2024). This is consistent with the trend for intermittent fires to increase the prevalence of wild *Sorghum* in the Katherine region of the Northern Territory (Norman 1969). Unexpectedly, in *S. timorense*, HCN potential was highest in the sheath, especially in accession St1 (209.7 μg g^−1^ under the control treatment). The ecological implications of storing more cyanogenic glucosides in sheaths than in leaves or roots warrant further study as it may confer resistance to stem borers and other pests that attack the sheath of *S. bicolor* (Okosun et al. 2021).

No significant effects of drought on HCN potential were found in the wild study species. Previously, Cowan et al. (2020) found some species in which HCN potential changed under drought, such as in the sheaths of *S. amplum* and *S. matrarankense*, and the roots of *S. brachypodum* and *S. macrospermum*. However, no consistent trend emerged, and in most species, no significant differences were detected between treatments (Cowan et al. 2020). Ananda et al. (2022) also found no significant effect of drought on leaf HCN potential in *S. macrospermum*, although leaves are typically the least important dhurrin storage organ in Australian wild *Sorghum* species. *Sorghum bicolor* , by contrast, is highly responsive to stress, with HCN potential of the leaves and stems reaching levels toxic to grazing animals in times of chronic drought, osmotic stress, or irrigated with saline water (Fu et al. 2025; Gleadow et al. 2016; O'Donnell et al. 2013; Sohail et al. 2022). Overall, it appears that many Australian wild *Sorghum* populations do not up‐regulate dhurrin biosynthesis in response to drought. This result is unsurprising when viewed alongside the broader results of this study, with most wild accessions not exhibiting as much plasticity in response to drought. The ability of wild *Sorghum* to maintain low dhurrin concentrations during drought warrants further exploration, as it could uncover opportunities to create safer *S. bicolor* lines that do not have high HCN potentials when stressed.

We did detect significant genotype effects on HCN potential in the roots of the wild study species, most notably a higher HCN potential in Sp1 than Sp2, and interactive effects of biomass and treatment on sheath HCN potential in *S. timorense*. Myrans et al. (2024) had also found that mean HCN potential was higher in the roots of Sp1 than Sp2 (accessions P6 and P2 in Myrans et al. 2024, respectively), and higher in the sheaths of St1 than St2 (accessions T5 and T2 in Myrans et al. 2024, respectively), but they found no significant correlation between HCN potential and aridity overall. Together, these results suggest that, while HCN potential is a plastic trait in *S. bicolor* (Bjarnholt et al. 2018; Gleadow et al. 2021), in the three species of wild *Sorghum* assessed here, it is more dependent on genotype than water availability. In both instances, the accession from the more arid provenance had the higher HCN potential, possibly due to more arid‐adapted accessions typically exhibiting conservative growth strategies in which a greater proportion of resources are allocated to storage and defense (Coley et al. 1985). The wild relatives may shed light on how the concentration of cyanogenic glucosides is regulated in domesticated sorghum and help in the identification of transcription factors (Rosati et al. 2024) or possible epigenetic effects (Rosati, Quinn, et al. 2019).

### 4.3 Overall Trends Are Related to Species, Morphology, and Aridity of Source Populations

In this study, wild *Sorghum* accessions experienced only minor phenotypic changes in response to water stress. These accessions generally have traits typically associated with a low‐risk, slow growth strategy featuring narrow leaves, storage of dhurrin in roots, and high leaf phenolic concentrations. These traits are likely adaptations to harsh, arid conditions in northern Australia (Lazarides et al. 1991; Myrans et al. 2021). Their narrow leaves and small root networks also meant that soil moisture was not depleted quickly, even under the drought treatment, and was possibly maintained at a high enough level to not inflict severe stress on these species. Further study is required to uncover traits that vary consistently with aridity in wild *Sorghum* species, with relationships remaining somewhat unclear. Reciprocal transplant experiments and thorough field surveys would offer valuable insight (Kawecki and Ebert 2004). However, when planning such studies, challenges arise from the extreme remoteness and inhospitable conditions of most wild *Sorghum* habitats. It would also be interesting to test the response of these species to longer‐term droughts, for example, that last until maturity, as this could uncover clearer relationships between phenotype and water deficiency.

For *S. bicolor* , there were multiple traits that experienced a significant biomass × treatment effect. Like many domesticated cereal crops, *S. bicolor* has fewer and broader leaves than its wild congeners. Its large biomass and fast growth make it susceptible to changes in the watering regime, with four of the measured traits significantly covarying with biomass under the different treatments, and a further two traits being significantly affected by the treatment. These effects were possibly exacerbated by mean VWC frequently being lower in the drought pots of *S. bicolor* than some wild accessions, although these differences were rarely significant (Table S1). Sorghum is often lauded for its high drought tolerance, compared to other cereals, and is often used as a failsafe crop (Hossain et al. 2022). However, there remains scope to improve its aboveground dhurrin levels under drought conditions, and the wild species in this study represent potential genetic resources for achieving this.

## 5 Conclusions

Regarding our hypotheses, there was evidence of reduced biomass under the drought treatment, but this difference was only significant in *S. bicolor* . Treatment also significantly affected dhurrin concentrations in *S. bicolor* but not the wild species. Phenolic concentration was significantly affected by treatment in *S. stipoideum* and significantly covaried with biomass under the different treatments in *S. bicolor* . Within the wild study species, there was little evidence of genotype × environment interactions in the form of plasticity. Nonetheless, some inherent intraspecific differences were observed between accessions, such as in dhurrin concentrations of *S. plumosum* and *S. stipoideum* . Most of the crop wild relatives of sorghum are native to Australia and remain understudied. These species are likely to provide useful insight into novel ways to create sorghum crops that are more resilient to the increasingly hot and dry conditions in much of the world, while also remaining safer for cattle grazing with lower dhurrin levels. More research is needed to quantify and characterize the high level of genotypic and phenotypic diversity within the genus.

## Conflicts of Interest

The authors declare no conflicts of interest.

## Supporting information


Data S1.

## Data Availability

The data that support the findings of this study are openly available in Monash Bridges at https://doi.org/10.26180/28406183.
