# Supplementary material for: Genotype × Environment Effects in Three Wild Relatives of Sorghum From Australia
Source: Plant Environ Interact. 2025 Jun 6;6(3):e70065. doi: 10.1002/pei3.70065 (PMC12142431; doi:10.1002/pei3.70065)
Supplement: Supplementary file 1 — Data S1. [file PEI3-6-e70065-s001.docx]

Table S1. Mean volumetric water content (VWC; %) readings ± SEM for each genotype × treatment group (n = 2-7), measured twice a week for 6.5 weeks. Within timepoints, results of two-way ANOVAs are shown, with significant *P* values shown in bold. Values with different letters are significantly different (*P* < 0.05)

| Genotype (accession code) | Treatment | Date | | | | | | | | | | | | |
| --- | --- | --- | --- | --- | --- | --- | --- | --- | --- | --- | --- | --- | --- | --- |
|  |  | 3/03/2023 | 6/03/2023 | 10/03/2023 | 13/03/2023 | 17/03/2023 | 20/03/2023 | 24/03/2023 | 27/03/2023 | 31/03/2023 | 3/04/2023 | 7/04/2023 | 10/04/2023 | 14/04/2023 |
| Sp1 | Control | 21.9 + 1.6 | 18.3 + 1.4^ab^ | 21.4 ± 0.8 | 20.9 ± 1.1^a^ | 19.8 ± 1.0 | 19.4 ± 2.0^a^ | 23.0 ± 0.2^a^ | 22.5 ± 2.0^a^ | 23.6 ± 0.6^a^ | 19.3 ± 1.0^a^ | 23.6 ± 0.7^a^ | 20.9 ± 1.2^a^ | 20.4 ± 1.9^ab^ |
|  | Drought | 22.2 + 1.5 | 17.7 + 1.7^ab^ | 21.4 ± 1.1 | 19.9 ± 1.2^a^ | 19.8 ± 1.4 | 10.9 ± 0.6^b^ | 10.1 ± 0.9^b^ | 8.8 ± 0.4^b^ | 8.8 ± 1.4^b^ | 2.2 ± 1.1^b^ | 7.7 ± 0.4^b^ | 7.2 ± 1.0^b^ | 6.8 ± 1.1^c^ |
| Sp2 | Control | 21.7 + 1.6 | 19.4 + 0.6^ab^ | 21.2 ± 1.0 | 19.8 ± 1.2^a^ | 19.9 ± 1.1 | 22.5 ± 1.0^a^ | 23.2 ± 1.1^a^ | 20.3 ± 1.0^a^ | 22.3 ± 1.0^a^ | 21.0 ± 1.0^a^ | 22.8 ± 0.7^a^ | 20.1 ± 1.7^a^ | 20.8 ± 1.2^ab^ |
|  | Drought | 25.3 + 1.3 | 22.9 + 0.9^ab^ | 22.1 ± 1.0 | 23.6 ± 1.0^a^ | 22.4 ± 1.0 | 11.4 ± 0.4^b^ | 9.5 ± 0.3^b^ | 8.9 ± 0.4^b^ | 5.6 ± 0.5^b^ | 7.1 ± 0.4^b^ | 7.3 ± 0.5^b^ | 5.5 ± 0.4^b^ | 6.6 ± 0.6^c^ |
| Ss1 | Control | 21.9 + 2.1 | 18.2 + 0.8^ab^ | 21.4 ± 0.4 | 20.7 ± 1.4^a^ | 19.9 ± 0.8 | 21.2 ± 1.7^a^ | 22.6 ± 0.8^a^ | 21.9 ± 1.1^a^ | 21.4 ± 0.7^a^ | 20.2 ± 1.1^a^ | 24.0 ± 1.0^a^ | 20.7 ± 1.2^a^ | 20.3 ± 1.1^ab^ |
|  | Drought | 23.5 + 0.8 | 21.1 + 0.9^ab^ | 21.4 ± 0.8 | 24.5 ± 0.8^a^ | 21.1 ± 1.2 | 10.5 ± 0.6^b^ | 9.9 ± 0.6^b^ | 8.3 ± 0.5^b^ | 8.5 ± 0.2^b^ | 7.9 ± 0.8^b^ | 7.8 ± 0.9^b^ | 7.1 ± 0.6^b^ | 6.6 ± 0.7^c^ |
| Ss2 | Control | 25.2 + 1.7 | 21.9 + 1.2^ab^ | 24.7 ± 1.0 | 22.7 ± 1.4^a^ | 22.2 ± 1.4 | 22.8 ± 2.0^a^ | 25.1 ± 0.9^a^ | 22.8 ± 0.8^a^ | 22.4 ± 0.9^a^ | 22.4 ± 1.7^a^ | 25.6 ± 0.7^a^ | 18.6 ± 1.0^a^ | 25.8 ± 2.2^a^ |
|  | Drought | 23.6 + 0.9 | 19.1 + 1.2^ab^ | 21.1 ± 1.6 | 20.4 ± 0.8^a^ | 20.6 ± 1.1 | 11.1 ± 0.3^b^ | 10.2 ± 0.4^b^ | 7.9 ± 0.4^b^ | 8.1 ± 0.3^b^ | 7.6 ± 0.4^b^ | 8.3 ± 0.5^b^ | 7.0 ± 0.5^b^ | 6.7 ± 0.5^c^ |
| St1 | Control | 23.3 + 2.0 | 20.9 + 1.8^ab^ | 20.4 ± 1.3 | 21.3 ± 1.1^a^ | 20.1± 1.1 | 21.0 ± 1.2^a^ | 23.1 ± 1.7^a^ | 21.4 ± 1.6^a^ | 22.0 ± 1.0^a^ | 21.6 ± 1.3^a^ | 22.5 ± 1.9^a^ | 19.8 ± 0.8^a^ | 18.8 ± 2.0^ab^ |
|  | Drought | 20.8 + 5.1 | 15.5 + 3.4^ab^ | 17.9 ± 1.9 | 21.0 ± 0.5^a^ | 22.1 ± 3.9 | 10.1 ± 0.6^b^ | 9.1 ± 0.1^b^ | 7.7 ± 1.2^b^ | 7.0 ± 0.9^b^ | 6.5 ± 0.2^b^ | 5.0 ± 1.0^bc^ | 4.9 ± 0.1^b^ | 5.1 ± 1.7^c^ |
| St2 | Control | 21.1 + 0.9 | 17.4 + 0.8^b^ | 21.3 ± 0.8 | 19.3 ± 1.1^a^ | 18.1 ± 0.7 | 20.5 ± 0.9^a^ | 22.2 ± 1.0^a^ | 18.7 ± 1.2^a^ | 20.0 ± 0.6^a^ | 19.8 ± 1.1^a^ | 22.3 ± 0.6^a^ | 20.9 ± 1.3^a^ | 18.3 ± 1.3^b^ |
|  | Drought | 23.9 + 0.9 | 20.6 + 0.9^a^ | 21.3 ± 0.8 | 20.6 ± 0.9^a^ | 19.9 ± 0.6 | 10.9 ± 0.6^b^ | 9.9 ± 0.4^b^ | 8.7 ± 0.6^b^ | 7.9 ± 0.4^b^ | 6.3 ± 0.6^b^ | 7.6 ± 0.3^b^ | 5.3 ± 0.8^b^ | 6.6 ± 1.2^c^ |
| Sb | Control | 20.9 ± 2.0 | 19.3 + 0.9^ab^ | 21.3 ± 1.5 | 21.4 ± 0.8^a^ | 21.4 ± 1.5 | 21.0 ± 1.0^a^ | 24.2 ± 1.0^a^ | 20.9 ± 1.1^a^ | 20.4 ± 0.7^a^ | 22.2 ± 1.6^a^ | 23.3 ± 1.1^a^ | 22.3 ± 1.1^a^ | 23.9 ± 1.4^ab^ |
|  | Drought | 23.4 ± 1.1 | 20.4 + 1.5^ab^ | 21.8 ± 0.7 | 21.2 ± 0.7^a^ | 21.2 ± 0.9 | 11.6 ± 0.6^b^ | 9.8 ± 0.7^b^ | 8.2 ± 0.7^b^ | 5.9 ± 1.0^b^ | 3.4 ± 1.1^b^ | 2.7 ± 1.2^c^ | 2.2 ± 1.3^b^ | 1.7 ± 1.3^c^ |
| ANOVA results | | | | | | | | | | | | | | |
| Treatment | df | 1 | 1 | 1 | 1 | 1 | 1 | 1 | 1 | 1 | 1 | 1 | 1 | 1 |
|  | F | 2.8 | 2.03 | 0.01 | 2.2 | 1.8 | 299.9 | 774.62 | 565.99 | 1088.41 | 588.89 | 1138.91 | 635.92 | 458.23 |
|  | *P* | 0.099 | 0.159 | 0.934 | 0.143 | 0.184 | **<0.001** | **<0.001** | **<0.001** | **<0.001** | **<0.001** | **<0.001** | **<0.001** | **<0.001** |
| Genotype | df | 6 | 6 | 6 | 6 | 6 | 6 | 6 | 6 | 6 | 6 | 6 | 6 | 6 |
|  | F | 0.55 | 1.38 | 1.37 | 1.16 | 1.34 | 0.7 | 0.83 | 0.84 | 3.21 | 0.88 | 3.9 | 0.66 | 2.26 |
|  | *P* | 0.766 | 0.234 | 0.239 | 0.339 | 0.254 | 0.651 | 0.551 | 0.543 | **0.008** | 0.515 | **0.002** | 0.684 | **0.048** |
| Genotype x Treatment | df | 6 | 6 | 6 | 6 | 6 | 6 | 6 | 6 | 6 | 6 | 6 | 6 | 6 |
|  | F | 0.98 | 3.44 | 1.49 | 2.45 | 0.88 | 0.45 | 0.6 | 1.48 | 0.84 | 2.13 | 2.3 | 3.01 | 4.09 |
|  | *P* | 0.448 | **0.005** | 0.195 | **0.034** | 0.513 | 0.844 | 0.728 | 0.199 | 0.545 | 0.061 | **0.044** | **0.011** | **0.001** |

Table S2. Results of testing for effects of genotype and treatment on biomass. Data for *S. plumosum*, *S. stipoideum* and *S. timorense* were analysed by two-way ANOVA. Data for S. bicolor were analysed by t-test. Significant *P* values are shown in bold

| Species | Genotype | | Treatment | | Genotype x Treatment | |
| --- | --- | --- | --- | --- | --- | --- |
|  | F | *P* | F | *P* | F | *P* |
| *S. plumosum*^†^ | 3.05 | 0.098 | 1.68 | 0.212 | 0.05 | 0.827 |
| *S. stipoideum*^†^ | 6.16 | **0.022** | 2.47 | 0.131 | 0.28 | 0.604 |
| *S. timorense* | 0.38 | 0.546 | 0.63 | 0.437 | 0.02 | 0.884 |
| *S. bicolor* | N/A | N/A | 2.25 | **0.048** | N/A | N/A |

^†^Data were log-transformed for this species.

Table S3. Results of ANCOVA analyses testing the effects of genotype, treatment and biomass on seven phenotypic parameters in *S. plumosum*. Significant *P* values are shown in bold

| Parameter | ANCOVA result | Effect | | | | | |
| --- | --- | --- | --- | --- | --- | --- | --- |
|  |  | Genotype | Biomass | Treatment | Genotype × biomass | Biomass × treatment | Genotype × treatment |
| Root:shoot ratio | df | 1 | 1 | 1 | 1 | 1 | 1 |
|  | F | 0.418 | 1.608 | 3.11 | 1.057 | 0.011 | 0.471 |
|  | *P* | 0.528 | 0.224 | 0.098 | 0.32 | 0.916 | 0.503 |
| Total [chlorophyll] | df | 1 | 1 | 1 | 1 | 1 | 1 |
|  | F | 0 | 1.17 | 1.114 | 1.981 | 0 | 1.641 |
|  | *P* | 0.987 | 0.297 | 0.308 | 0.18 | 0.987 | 0.22 |
| Chlorophyll a:b ratio^†^ | df | 1 | 1 | 1 | 1 | 1 | 1 |
|  | F | 0.804 | 1.646 | 2.326 | 1.785 | 0.13 | 3.875 |
|  | *P* | 0.384 | 0.219 | 0.148 | 0.201 | 0.724 | 0.068 |
| Leaf [phenolics] | df | 1 | 1 | 1 | 1 | 1 | 1 |
|  | F | 30.135 | 0.579 | 0.138 | 3.62 | 0.528 | 2.428 |
|  | *P* | **<0.001** | 0.458 | 0.715 | 0.076 | 0.479 | 0.14 |
| Leaf HCN potential^†^ | df | 1 | 1 | 1 | 1 | 1 | 1 |
|  | F | 14.683 | 0.14 | 0.98 | 0.243 | 0.79 | 0.504 |
|  | *P* | **0.002** | 0.713 | 0.338 | 0.629 | 0.388 | 0.489 |
| Sheath HCN potential^†^ | df | 1 | 1 | 1 | 1 | 1 | 1 |
|  | F | 4.551 | 1.935 | 0.17 | 0.736 | 0.01 | 1.28 |
|  | *P* | **0.049** | 0.185 | 0.686 | 0.405 | 0.922 | 0.276 |
| Root HCN potential^†^ | df | 1 | 1 | 1 | 1 | 1 | 1 |
|  | F | 7.497 | 0.054 | 0.456 | 0 | 0.29 | 0.072 |
|  | *P* | **0.015** | 0.82 | 0.51 | 0.996 | 0.598 | 0.793 |

^†^Data were log-transformed for this parameter.

Table S4. Results of ANCOVA analyses testing the effects of genotype, treatment and biomass on seven phenotypic parameters in *S. stipoideum*. Significant *P* values are shown in bold

| Parameter | ANCOVA result | Effect | | | | | |
| --- | --- | --- | --- | --- | --- | --- | --- |
|  |  | Genotype | Biomass | Treatment | Genotype × biomass | Biomass × treatment | Genotype × treatment |
| Root:shoot ratio^†^ | df | 1 | 1 | 1 | 1 | 1 | 1 |
|  | F | 1.549 | 0.855 | 0.576 | 0.47 | 0.029 | 2.376 |
|  | *P* | 0.229 | 0.368 | 0.458 | 0.502 | 0.868 | 0.141 |
| Total [chlorophyll] | df | 1 | 1 | 1 | 1 | 1 | 1 |
|  | F | 0.048 | 4.936 | 3.943 | 0.336 | 0.001 | 0.079 |
|  | *P* | 0.829 | **0.039** | 0.063 | 0.569 | 0.983 | 0.782 |
| Chlorophyll a:b ratio^†^ | df | 1 | 1 | 1 | 1 | 1 | 1 |
|  | F | 0.585 | 1.268 | 0.146 | 1 | 0.249 | 0.676 |
|  | *P* | 0.455 | 0.275 | 0.707 | 0.331 | 0.624 | 0.422 |
| Leaf [phenolics] | df | 1 | 1 | 1 | 1 | 1 | 1 |
|  | F | 0.665 | 0.567 | 5.406 | 0.337 | 1.837 | 0.622 |
|  | *P* | 0.425 | 0.461 | **0.032** | 0.569 | 0.192 | 0.441 |
| Leaf HCN potential^†^ | df | 1 | 1 | 1 | 1 | 1 | 1 |
|  | F | 6.554 | 0.773 | 0.216 | 0.601 | 0.558 | 0.249 |
|  | *P* | **0.02** | 0.391 | 0.647 | 0.448 | 0.465 | 0.624 |
| Sheath HCN potential^†^ | df | 1 | 1 | 1 | 1 | 1 | 1 |
|  | F | 3.285 | 0.047 | 0.117 | 0.189 | 1.008 | 0.169 |
|  | *P* | 0.087 | 0.832 | 0.736 | 0.669 | 0.329 | 0.686 |
| Root HCN potential^†^ | df | 1 | 1 | 1 | 1 | 1 | 1 |
|  | F | 0.586 | 0.69 | 0.131 | 0.465 | 0.261 | 0.587 |
|  | *P* | 0.454 | 0.417 | 0.722 | 0.504 | 0.616 | 0.454 |

^†^Data were log-transformed for this parameter.

Table S5. Results of ANCOVA analyses testing the effects of genotype, treatment and biomass on seven phenotypic parameters in *S. timorense*. Significant *P* values are shown in bold

| Parameter | ANCOVA result | Effect | | | | | |
| --- | --- | --- | --- | --- | --- | --- | --- |
|  |  | Genotype | Biomass | Treatment | Genotype × biomass | Biomass × treatment | Genotype × treatment |
| Root:shoot ratio | df | 1 | 1 | 1 | 1 | 1 | 1 |
|  | F | 4.316 | 0.209 | 2.7 | 0.031 | 0.915 | 0.606 |
|  | *P* | 0.055 | 0.654 | 0.121 | 0.863 | 0.354 | 0.448 |
| Total [chlorophyll] | df | 1 | 1 | 1 | 1 | 1 | 1 |
|  | F | 1.884 | 0.118 | 0.052 | 2.172 | 0.016 | 0.85 |
|  | *P* | 0.19 | 0.737 | 0.822 | 0.161 | 0.9 | 0.371 |
| Chlorophyll a:b ratio | df | 1 | 1 | 1 | 1 | 1 | 1 |
|  | F | 0.412 | 0.448 | 0.307 | 2.938 | 0.108 | 0.018 |
|  | *P* | 0.531 | 0.513 | 0.588 | 0.107 | 0.748 | 0.9 |
| Leaf [phenolics] | df | 1 | 1 | 1 | 1 | 1 | 1 |
|  | F | 1.468 | 0.817 | 1.621 | 1.088 | 2.287 | 2.046 |
|  | *P* | 0.245 | 0.38 | 0.222 | 0.313 | 0.151 | 0.173 |
| Leaf HCN potential^†^ | df | 1 | 1 | 1 | 1 | 1 | 1 |
|  | F | 0.37 | 0.94 | 0.04 | 0.395 | 1.25 | 0.037 |
|  | *P* | 0.552 | 0.348 | 0.844 | 0.539 | 0.281 | 0.849 |
| Sheath HCN potential^†^ | df | 1 | 1 | 1 | 1 | 1 | 1 |
|  | F | 29.504 | 0.486 | 3.66 | 2.481 | 5.036 | 0.037 |
|  | *P* | **<0.001** | 0.496 | 0.075 | 0.136 | **0.04** | 0.85 |
| Root HCN potential | df | 1 | 1 | 1 | 1 | 1 | 1 |
|  | F | 0.217 | 0.096 | 0.598 | 0.017 | 0.71 | 0.089 |
|  | *P* | 0.648 | 0.761 | 0.452 | 0.899 | 0.413 | 0.769 |

^†^Data were log-transformed for this parameter.

Table S6. Results of ANOVA analyses testing the effects of treatment and biomass on seven phenotypic parameters in *S. bicolor*. Significant *P* values are shown in bold

| Parameter | ANCOVA result | Effect | | |
| --- | --- | --- | --- | --- |
|  |  | Biomass | Treatment | Biomass × treatment |
| Root:shoot ratio | df | 1 | 1 | 1 |
|  | F | 3.461 | 17.873 | 13.944 |
|  | *P* | 0.1 | **0.003** | **0.006** |
| [Total chlorophyll] | df | 1 | 1 | 1 |
|  | F | 2.128 | 10.012 | 4.466 |
|  | *P* | 0.183 | **0.013** | 0.068 |
| Chlorophyll a:b ratio | df | 1 | 1 | 1 |
|  | F | 1.737 | 17.497 | 14.821 |
|  | *P* | 0.224 | **0.003** | **0.005** |
| Leaf [phenolics] | df | 1 | 1 | 1 |
|  | F | 2.667 | 7.842 | 7.84 |
|  | *P* | 0.141 | **0.023** | **0.023** |
| Leaf HCN potential | df | 1 | 1 | 1 |
|  | F | 7.536 | 7.668 | 16.425 |
|  | *P* | **0.025** | **0.024** | **0.004** |
| Sheath HCN potential | df | 1 | 1 | 1 |
|  | F | 0.017 | 9.568 | 3.911 |
|  | *P* | 0.898 | **0.015** | 0.083 |
| Root HCN potential^†^ | df | 1 | 1 | 1 |
|  | F | 4.651 | 0.107 | 0.001 |
|  | *P* | 0.063 | 0.752 | 0.971 |

^†^Data were log-transformed for this parameter.


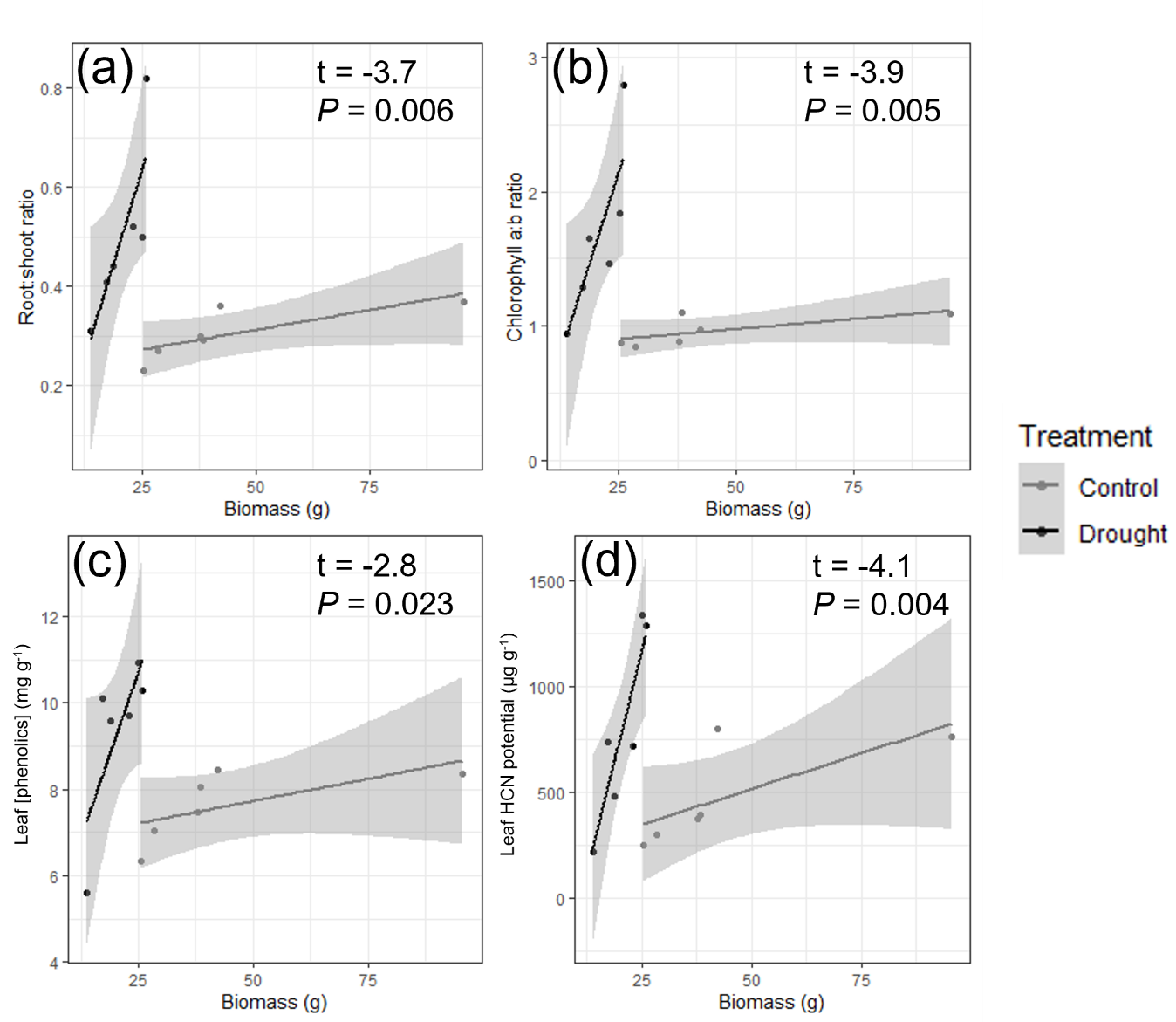


Figure S1. Separate slopes analyses of significant biomass × treatment interaction effects on (a) root:shoot ratio, (b) chlorophyll a:b ratio, (c) leaf [phenolics] and (d) leaf HCN potential in *S. bicolor*. Black points and lines represent the drought treatment, while grey points and lines represent the control treatment. Shading around trendlines represents the standard error. t and *P* values from the separate slopes analyses are displayed


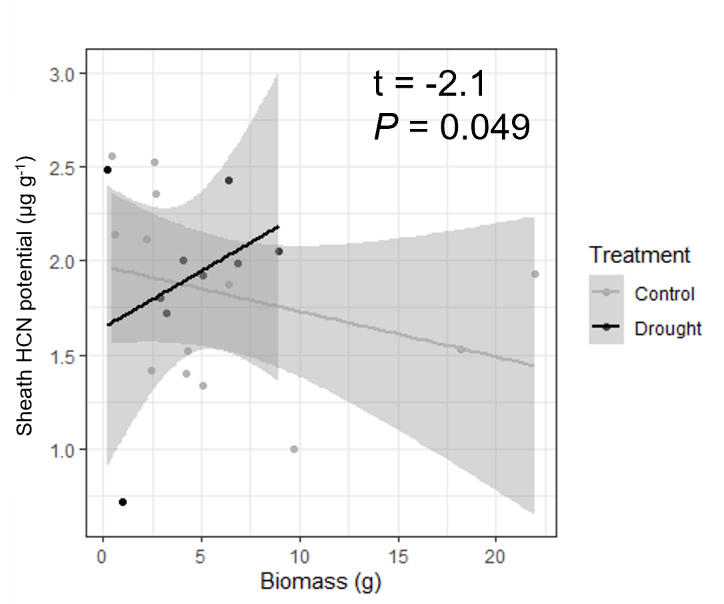


Figure S2. Separate slopes analysis of significant biomass × treatment interaction effects on sheath HCN potential in *S. timorense*. Black points and lines represent the drought treatment, while grey points and lines represent the control treatment. Shading around trendlines represents the standard error. t and *P* values from the separate slopes analysis are displayed
